# Hydrogen gas represses the progression of lung cancer via down-regulating CD47

**DOI:** 10.1042/BSR20192761

**Published:** 2020-04-28

**Authors:** Jinghong Meng, Leyuan Liu, Dongchang Wang, Zhenfeng Yan, Gang Chen

**Affiliations:** 1 Department of Rheumatology and Immunology, The Third Hospital of Hebei Medical University, Shijiazhuang, Hebei, China; 2 Department of Respiration, The Third Hospital of Hebei Medical University, Shijiazhuang, Hebei, China

**Keywords:** CD47, Hydrogen gas (H2), lung cancer, phagocytosis, tumor growth

## Abstract

Hydrogen gas (H_2_) has been identified to play an anti-tumor role in several kinds of cancers, but the molecular mechanisms remain largely unknown. In our previous study, our project group found that H_2_ could decrease the expression of CD47 in lung cancer A549 cells via the next-generation sequencing, indicating that CD47 might be involved in H_2_-mediated lung cancer repression. Therefore, the present study aimed to explore the effects of CD47 on H_2_-induced lung cancer repression. Western blotting and real-time PCR (RT-PCR) assays were used to detect the levels of proteins and mRNAs, respectively. Cell proliferation, invasion, migration and apoptosis were detected by using the cell counting kit-8 (CCK-8), Transwell chambers, wound healing and flow cytometry assays, respectively. The results showed that H_2_ treatment caused decreases in the expression levels of CD47 and cell division control protein 42 (CDC42) in a dose-dependent manner. Up-regulation of CD47 abolished H_2_ roles in promoting lung cancer cell apoptosis and repressing cell growth, invasion and migration in both A549 and H1975 cell lines. However, knockdown of CD47 enhanced H_2_ role in lung cancer inhibition. Moreover, we also observed that H_2_ treatment induced obvious inhibitions in the expression levels of CDC42 and CD47 in mice tumor tissues, as well as reinforced macrophage-mediated phagocytosis in A549 and H1975 cells. In conclusion, the current study reveals that H_2_ inhibits the progression of lung cancer via down-regulating CD47, which might be a potent method for lung cancer treatment.

## Introduction

Lung cancer is the most common malignant tumor and the main reason of cancer-related deaths all over the world. It is estimated that the newly diagnosed cases of lung cancer accounted for ∼12.9% among all new cases of tumors in 2012 [1]. Non-small cell lung cancer (NSCLC) accounts for approximately 85% of all lung cancer types, with a 5-year survival rate for patients at stage IV less than 1% [2]. Most patients with NSCLC will develop metastasis when they are first diagnosed [3,4], losing the perfect time for surgery. Therefore, it is essential to find new effective treatment strategies to improve the outcome of NSCLC patients.

Hydrogen gas (H_2_), as a kind of endogenous gas, has been identified not only to serve as a crucial energy source, but also exerts important physiological regulation roles [5]. Hydrogen molecules can enter into tissues and exert anti-inflammatory, antioxidant and anti-apoptotic roles [6]. As early as 1975, Dole et al. [7] put forward for the first time, that H_2_ had the ability to treat cancers. In our previous study, we found that H_2_ administration significantly repressed lung cancer A549 and H1975 cell proliferation, migration and invasion and induced cell apoptosis [8]. However, the molecular mechanisms still remain largely unknown.

CD47 is a transmembrane glycoprotein which is widely expressed in normal tissues and mediates a ‘self/don’t-eat-me’ signaling via repressing macrophage phagocytosis [9,10]. CD47 has been reported to be frequently up-regulated in multiple kinds of cancers, such as ovarian cancer [11], gastric cancer [12], breast cancer [13] and NSCLC [14]. Noticeably, it has been identified that CD47 induces the immunological evasion of cancer cells, and is considered as a potential target for cancer treatment [15,16]. Using sequencing technique, we found that H_2_ induced a significant reduction in CD47 expression in lung cancer A549 cells, but weather CD47 is involved in H_2_-mediated inhibition in lung cancer progression still remains unclear.

In the current study, we aimed to elucidate the roles of CD47 in H_2_-mediated inhibition of lung cancer progression though carrying out both *in vivo* and *in vitro* experiments.

## Materials and methods

### Cell lines and culture

Two lung cancer cell lines A549 and H1975 cells were purchased from American Type Culture Collection (ATCC; Manassas, VA, U.S.A.). A549 cells were cultured in F-12K Medium (Gibco, Thermo Fisher Scientific, MA, U.S.A.), supplemented with 10% fetal bovine serum (FBS; Gibco). H1975 cells were grown in RPMI-1640 medium (Gibco) supplemented with 10% FBS. All cell lines were maintained in an incubator at 37°C with 5% CO_2_.

### H_2_ treatment

A549 and H1975 cells were cultured in 20, 40 and 60% H_2_ with the help of hydrogen machine (Shanghai Nanobubble Technology Co., Ltd., Shanghai, China) for different times, and 5% CO_2_ rved as the negative control.

### Cell transfection

Vectors used to overexpress CD47 (OE-CD47) and the small interfering RNAs (siRNAs) used to silence CD47 (si-CD47), as well as their negative control vectors (OE-NC, si-NC) were all obtained from GenePharma (Shanghai, China). A549 and H1975 cells were transfected with these vectors using the Lipofectamine 2000 transfected reagent (Invitrogen, Carlsbad, CA, U.S.A.) referring to the manufacturer’s instructions.

### Quantitative real-time PCR analysis

The total RNAs were extracted from cells using the RNApure Tissue & Cell Kit (DNase I) in accordance with the manufacturer’s instructions (CWBio, Beijing, China). Then, a total of 1 μg RNA from each sample was subjected to cDNA reverse transcription and real-time PCR (RT-PCR) using the Quant One Step RT-PCR kit (TIANGEN, Beijing, China) on Bio-Rad detection system (Bio-Rad, Hercules, CA) after RNA quantification via using an ND-1000 NanoDrop Spectrophotometer (NanoDrop Technologies, Inc., Wilmington, Delaware). GAPDH expression level serves as an internal reference. Primers were listed as follows,

CD47: forward (F) 5′-CGGCGTGTATACCAATGC-3′; Reverse (R) 5′-TTTGAATGCATTAAGGGGTTCCT-3′;

GAPDH: F 5′-CCACTAGGCGCTCACTGTTCTC-3′; R 5′-ACTCCGACCTTCACCTTCCC-3′.

### Western blotting assay

Total proteins were extracted from cells using the RIPA lysis buffer (Beyotime Biotechnology, Shanghai, China) supplemented with protease inhibitor for 30 min at 4°C. Following quantification with a BCA Protein Kit (Bio- Rad Laboratories, CA, U.S.A.), 30 μg proteins from each sample were separated by 10% polyacrylamide gels, and were transferred to the polyvinylidene difluoride membranes (Millipore, Billerica, MA, U.S.A.). The membranes were then incubated with 5% non-fat milk and probed with the primary antibodies overnight at 4°C, including Bcl-2 (1:1000 dilution; No. #3498, Cell Signaling Technology, MA, U.S.A.), cleaved caspase3 (1:1000 dilution; No. 9661, Cell Signaling Technology), caspase3 (1:1000 dilution; No. #9662, Cell Signaling Technology), cleaved PARP (1:1000 dilution; #5625, Cell Signaling Technology), PARP (1:1000 dilution; #9532, Cell Signaling Technology), CD47 (1:1000 dilution; No. #63000, Cell Signaling Technology), cell division control protein 42 (CDC42) (1:1000 dilution; No. #2462, Cell Signaling Technology), β-actin (1:2000 dilution; No. #4970, Cell Signaling Technology). Following incubation with the corresponding second antibodies (Santa Cruz Biotechnology, Dallas, TX, U.S.A.), the protein expressions were examined on a Western blotting imaging and quantitative system (Bio-Rad) after incubation with chemiluminescent ECL reagent (Millipore). The quantification of proteins was carried out by using the ImageJ software (National Institutes of Health) after background of subtraction, with β-actin expression as an internal reference.

### Cell counting kit-8 assay

Cell counting kit-8 (CCK-8) assay was used to detect cell proliferation. In brief, A549 and H1975 cells were seeded in 96-well plates at a density of 3 × 10^3^ cells/well and cultured at 37°C overnight, then the cells were given H_2_ treatment and/or cell transfection. Following incubation at 37°C for the indicated times, the cell culture medium was replaced with 10 μl of CCK-8 reagent (Beyotime, Beijing, China) and 90 μl fresh medium, and the cells were incubated at 37°C for another 4 h. The absorbance at 450 nm was measured with a plate reader (model 680; Bio-Rad, Hertfordshire, U.K.).

### Flow cytometry assay

Flow cytometry assay was carried out for cell apoptosis assessment. In brief, different treated A549 and H1975 cells were harvested and subjected to apoptosis test using Annexin V (FITC)/propidium iodide (PI) Apoptosis Detection Kits (Dojindo, Japan). Cell apoptosis rate was detected by using flow cytometry (Beckman Coulter, CA, U.S.A.) and analyzed using FlowJo 7.6 software (FlowJo LLC, Ashland, OR, U.S.A.).

### Wound healing assay

Wound healing assay was used to detect cell migration ability. Briefly, A549 and H1975 cells were plated in six-well plates at a concentration of 5 × 10^5^ cells/ml and incubated at 37°C overnight, followed by different cell transfections. The wounds were made using 20 μl pipette tips when cell confluence reached at 100%, and the gloating cells were removed via PBS washing. Then, the cells were cultured at 37°C with 60% H_2_ administration or not. Images of cells movement to the scratch area were taken every 6–12 h using a microscope.

### Transwell chamber assay

Transwell chambers with 8-μm polycarbonate filters (BD Bioscience, San Diego, CA, U.S.A.) were applied for cell invasion assessment. In procedure, chambers were coated with Matrigel on the lower side. Then, approximately 2 × 10^5^ of A549 or H1975 cells resuspended with FBS-free medium were seeded in the upper chamber, while 600 μl medium supplemented with 20% FBS were added into the lower chamber. Following 48 h of incubation at 37°C with 5% CO_2_ or 60% H_2_, cells in the top of the membranes were removed using cotton buds and cells at the bottom of the membrane were fixed and stained with 0.2% Crystal Violet (Solarbio, Beijing, China). The stained cells were counted under a light microscope (magnification: 200×) to assess cell invasiveness.

### *In vivo* experiment

Animal experiment was performed in the Third Hospital of Hebei Medical University by Wang et al. [8] in accordance with National Institute of Health’s Guidelines for the Care and Use of Laboratory Animals, and was given permission by Animal Care and Research Committee of the Third Hospital of Hebei Medical University. Four-week male BALB/c athymic nude mice (Beijing Vital River Laboratory Animal Technology, Beijing, China) were fed in specific pathogen-free conditions. A total of 1 × 10^7^ A549 cells suspended in 200 μl PBS were injected into the BALB/c mice, which were then divided into three groups when the tumors were grown 3–4 mm in diameter, those were H_2_ group, *cis*-platinum group and control group. Mice in H_2_ group were given 60% H_2_ inhaling for 2 h every day, mice in *cis*-platinum group were given *cis*-platinum (10 mg/kg body weight) [17] intraperitoneal injection. H_2_ and *cis*-platinum treatments were kept for 4 weeks. Then, the mice were killed via cervical dislocation. Atmosphere was used as a control of 60% H_2_ and the same amount of saline was served as a control for *cis*-platinum.

### *In vitro* phagocytosis assay

The ability of macrophage-mediated phagocytosis was measured according to a previous study [18]. In briefly, 1 × 10^5^ macrophages were seeded into the culture dishes with glass bottom. Then, A549 and H1975 cells were labeled with 20 μM CFDA-SE using a Vybrant CFDA-SE Cell Tracer Kit (Invitrogen), which were then inoculated into the macrophages culture. Macrophages were washed and then photographed with confocal microscope. The phagocytic index was evaluated by the number of phagocytosed carboxy fluoresce in succinimidyl ester (CFSE)-positive cells per 100 macrophages.

### Hematoxylin and Eosin and immunohistochemistry staining

Mice tissue samples from H_2_, *cis*-platinum and control groups were contributed by Wang et al. [8]. The cancer tissues were removed and subjected to Hematoxylin and Eosin (HE) and immunohistochemistry (IHC) staining to evaluate the histopathology and the protein expressions of CD47 and CDC42 referring to the following procedures. In brief, the paraffin-embedded cancer tissues were cut into 4-μm sections, followed by being sectioned, dewaxed and hydrated and incubation with 3% H_2_O_2_ for 10 min and antigen retrieval using Tris-EDTA. The sections were then sealed with 5% goat serum and subsequently probed with anti-CD47 (1:200 dilution; No. ab175388, Abcam, Cambridge, MA, U.S.A.) or anti-CDC42 (1:150 dilution; No. ab64533, Abcam) antibody overnight at 4°C and the corresponding secondary antibody. After being washed with PBS for three times, the sections were incubated with chromogen 3,3′-diaminobenzidinetetrachloride (DAB) (Serva, Heidelberg, Germany), which was served as a substrate.

### Statistical analysis

Each experiment in the current study was performed in triplicate. Statistical significance comparison between two groups and multiple groups was performed by using Student’s *t* test and one-way ANOVA, respectively. Data analysis was performed by using GraphPad Prism (version 6.0, La Jolla, CA, U.S.A.). *P*<0.05 was considered as statistically significant.

## Results

### H_2_ administration represses the malignant transformation of lung cancer cells

**Figure 1:**
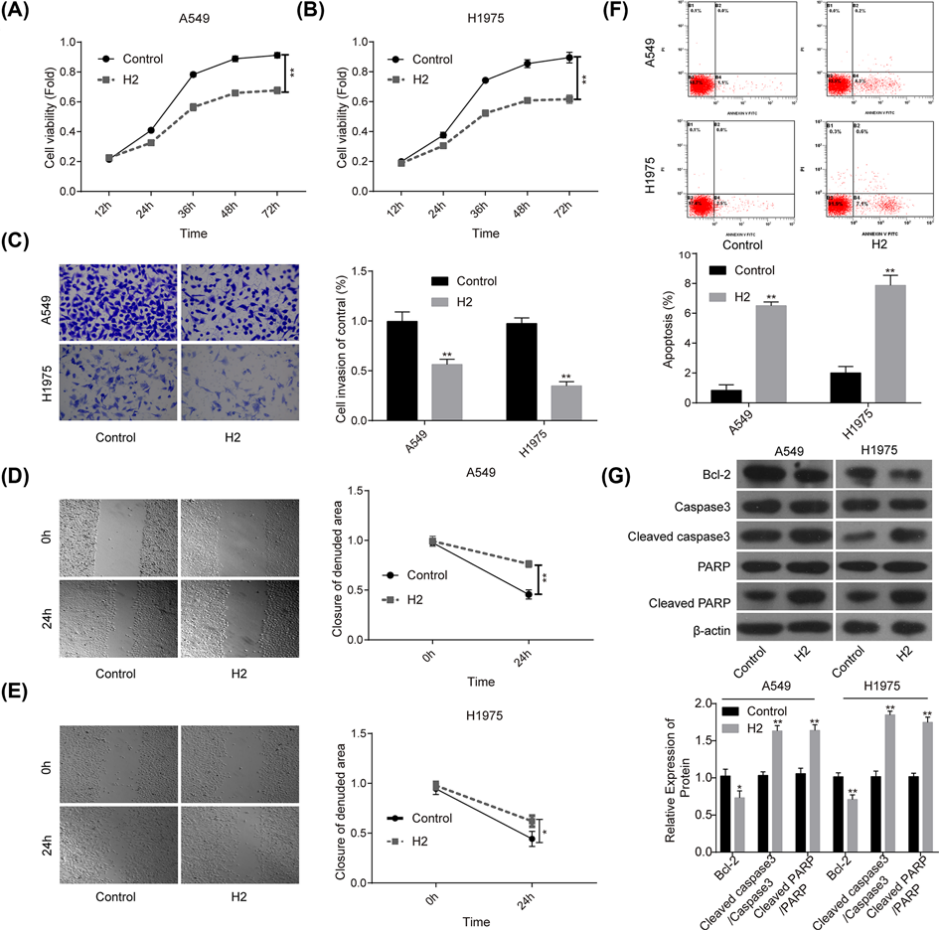
H_2_ treatment inhibited cell proliferation, migration and invasion and induced cell apoptosis in A549 and H1975 cells CCK-8 assay was used to detect cell proliferation after cells were treated with 60% H_2_ for 12, 24, 36, 48 and 72 h in (**A**) A549 and (**B**) H1975 cells. (**C**) Cell invasion ability was determined by using the Transwell chambers after 48 h of cell treatment with 60% H_2_. (**D,E**) Wound healing assay was used to assess cell migration ability after 24 h of cell treatment with 60% H_2_. (**F**) The effects of H_2_ treatment on cell apoptosis were determined by flow cytometry assay in A549 and H1975 cells. (**G**) Western blotting was carried out to detect the expressions of Bcl-2, cleaved caspase3, caspase3, cleaved PARP and PARP after 48 h of cell treatment with 60% H_2_ (*P<0.05, **P<0.01).

First, we explored the roles of 60% H_2_ administration in the progression of lung cancer *in vitro*. Compared with the control group, cell proliferation (Figure 1A,B), invasion (Figure 1C) and migration (Figure 1D,E) abilities were all significantly repressed when cells were given 60% H_2_ treatment in both A549 and H1975 cell lines. In addition, cell apoptosis rates in both A549 and H1975 cells were obviously increased in H_2_ groups when compared with that of the control group (Figure 1F), accompanied with decreased expression of Bcl-2 and the increases in cleaved caspase3/caspase3 and cleaved PARP/PARP levels (Figure 1G). These results suggested that H_2_ treatment could repress the progression of lung cancer *in vitro*.

### H_2_ treatment down-regulates the expression of CD47 and CDC42 in lung cancer

**Figure 2:**
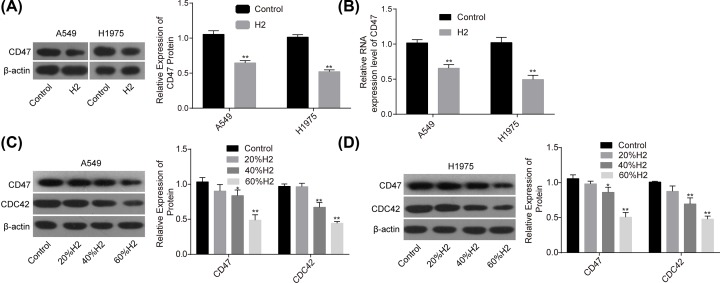
H_2_ treatment decreased the expressions of CD47 and CDC42 in A549 and H1975 cells After 48 h of cell treatment with H_2_, A549 and H1975 cells were collected and subjected to RNA and DNA extraction with the following detections. (**A,B**) The mRNA and protein levels of CD47 were detected by RT-PCR and Western blotting assays. (**C,D**) The protein expression levels of CD47 and CDC42 were detected by Western blotting after cell treatments with different concentrations of H_2_ (*P<0.05, **P<0.01).

Our research group previously revealed that H_2_ treatment could induce a significant decrease in CD47 expression [8], indicating that CD47 might be involved in H_2_-mediated lung cancer inhibition. To this end, we used Western blotting assay to explore the effect of H_2_ treatment on the expression of CD47. Compared with the control group, the mRNA and protein expression levels of CD47 were all significantly decreased when A549 and H1975 cells were treated with 60% H_2_ (Figure 2A,B). Moreover, H_2_ treatment decreased CD47 and CDC42 expressions in a dose-dependent manner in A549 (Figure 2C) and H1975 cells (Figure 2D). These results demonstrated that H_2_ negatively regulated CD47 and CDC42 expression in lung cancer.

### Knockdown of CD47 cooperates with H_2_ to repress the progression of lung cancer

**Figure 3:**
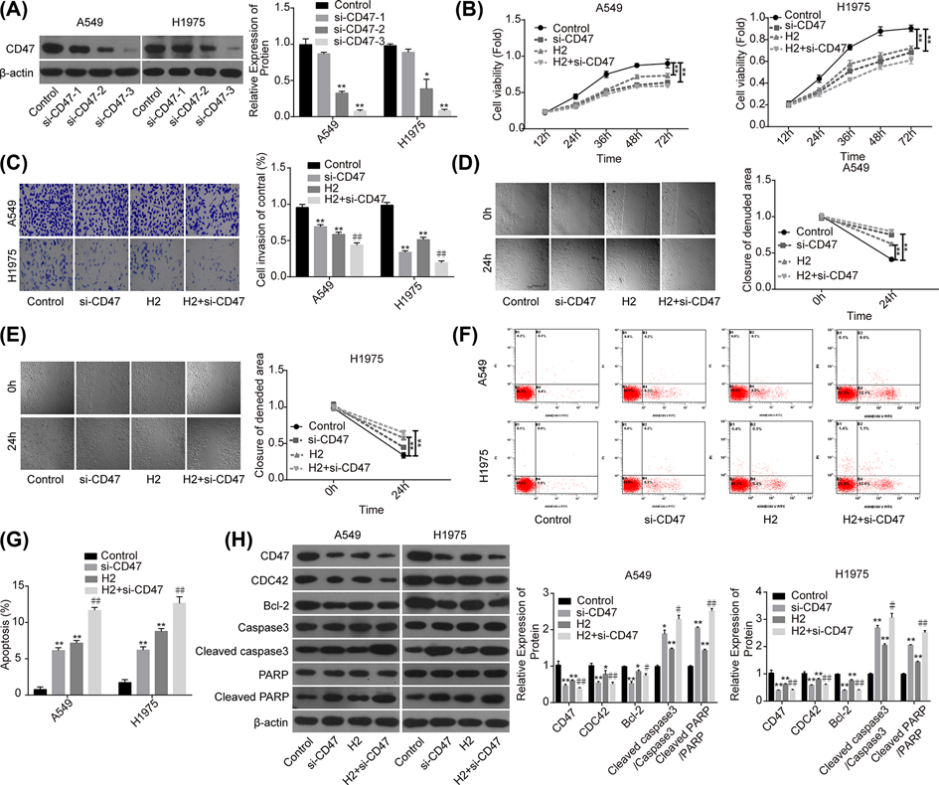
Knockdown of CD47 cooperated with H_2_ to repress the progression of lung cancer A549 and H1975 cells transfected with si-CD47 or si-NC were treated with 60% H_2_, then the following assays were performed. (**A**) Western blotting assay was used to detect the knockdown efficiency of si-CD47 in A549 and H1975 cells. (**B**) CCK-8 assay was used to detect cell proliferation. (**C**) Transwell chambers were applied for cell invasion assessment. (**D,E**) Wound healing assay was used to detect cell migration ability. (**F,G**) The effects of H_2_ treatment on the apoptosis of A549 and H1975 cells were determined by flow cytometry assay. (**H**) Western blotting was carried out to detect the expressions of CD47, CDC42, Bcl-2, cleaved caspase3, caspase3, cleaved PARP and PARP after 48 h of cell treatment with 60% H_2_ (*P<0.05, **P<0.01 vs. control group; ^#^*P*<0.05, ^##^*P*<0.01 vs. H_2_ group).

Then, we performed the loss-of-function assay to explore the role of CD47 in H_2_-mediated lung cancer repression. Compared with the si-NC group, the expression of CD47 was significantly decreased when A549 and H1975 cells were transfected with si-CD47, and si-CD47-3 showed the best knockdown efficiency (Figure 3A). Compared with the control group, cell proliferation (Figure 3B), invasion (Figure 3C) and migration (Figure 3D,E) capacities were all significantly decreased when the cells were transfected with si-CD47 or treated with 60% H_2_. In addition, both down-regulation of CD47 and H_2_ treatment induced a significant increase in cell apoptosis rate (Figure 3F,G), and decreases in the expressions of Bcl-2 and CDC42, as well as the increasein the levels of cleaved caspase3/caspase3 and cleaved PARP/PARP (Figure 3H). Noticeably, combination of si-CD47 with H_2_ treatment showed higher efficiency than si-CD47 or H_2_ treatment alone (Figure 3B–H). These findings illustrated that CD47 down-regulation could enhance H_2_ effect on the repression of lung cancer progression.

### Overexpression of CD47 impairs H_2_ role in repressing the progression of lung cancer

**Figure 4:**
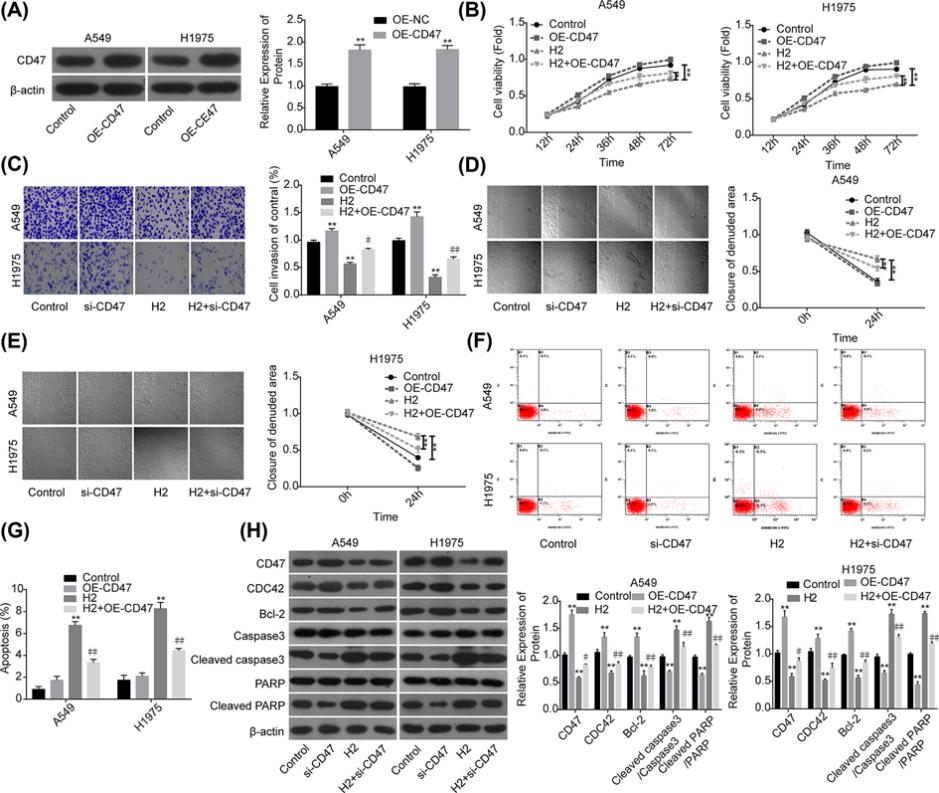
Overexpression of CD47 impaired H_2_ roles in repressing the progression of lung cancer A549 and H1975 cells transfected with OE-CD47 or OE-NC were treated with 60% H_2_, then the following assays were performed. (**A**) Western blotting assay was used to detect the protein expression of CD47 after A549 and H1975 cells were transfected with OE-CD47 or OE-NC. (**B**) CCK-8 assay was used to detect cell proliferation. (**C**) Transwell chambers were applied for cell invasion assessment. (**D,E**) Wound healing assay was used to detect cell migration ability. (**F,G**) The effects of H_2_ treatment on the apoptosis of A549 and H1975 cells were determined by flow cytometry assay. (**H**) Western blotting was carried out to detect the expressions of CD47, CDC42, Bcl-2, cleaved caspase3, caspase3, cleaved PARP and PARP after 48 h of cell treatment with 60% H_2_ (*P<0.05, **P<0.01 vs. control group; ^#^*P*<0.05, ^##^*P*<0.01 vs. H_2_ group).

To further reveal the role of CD47 in H_2_-mediated lung cancer inhibition, we also performed the gain-of-function assay. The expression of CD47 was apparently increased when A549 and H1975 cells were transfected with OE-CD47 (Figure 4A). To the opposite of H_2_ roles in lung cancer functions alteration, cell proliferation (Figure 4B), invasion (Figure 4C) and migration (Figure 4D,E) abilities were all obviously enhanced when cells were transfected with OE-CD47. Moreover, CD47 overexpression significantly inhibited cell apoptosis (Figure 4F,G) and increased the expression of Bcl-2 while decreased the expression levels of cleaved caspase3/caspase3 and cleaved PARP/PARP (Figure 4H). Furthermore, CD47 overexpression significantly abolished H_2_ roles in the inhibitions of cell growth, migration, invasion and Bcl-2 expression, as well as the promotions of cell apoptosis and the expressions of cleaved caspase3/caspase3 and cleaved PARP/PARP (Figure 4B–H). These results further confirmed that H_2_ treatment inhibited the progression of lung cancer via decreasing CD47 expression.

### H_2_ treatment represses the expressions of CD47 and CDC42 in lung cancer tissues from *in vivo* mice

**Figure 5:**
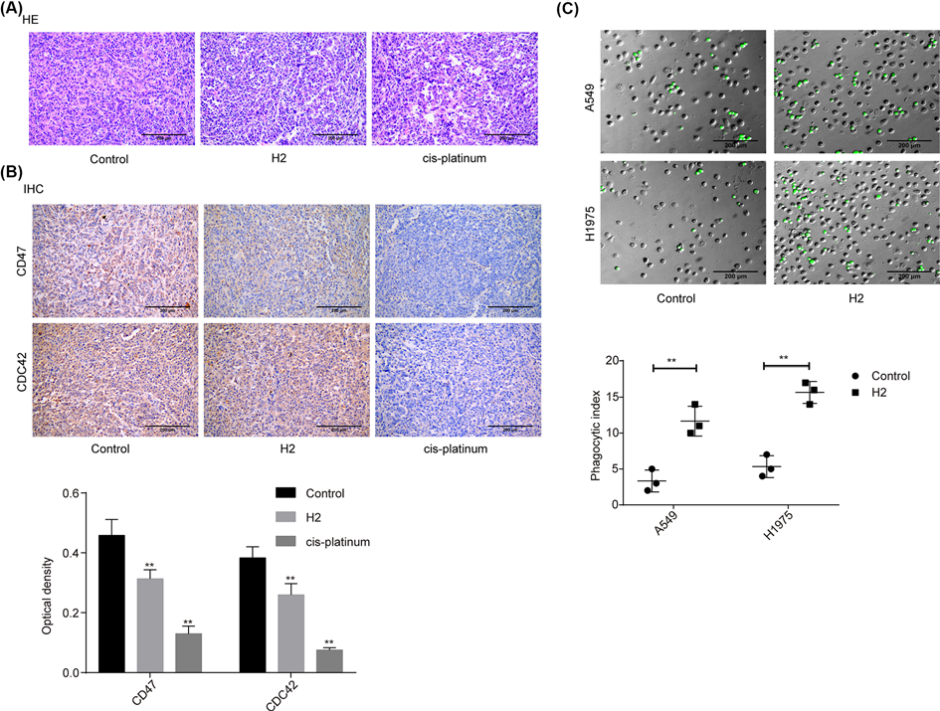
H_2_ treatment repressed the progression of lung cancer *in vivo* (**A**) HE staining was carried out to assess the histopathology of lung cancer tissues from different treated mice (scale bar = 200 μm). (**B**) IHC staining was used to evaluate the expressions of CD47 and CDC42 in lung cancer tissues from different treated mice (scale bar = 200 μm). (**C**) *In vitro* phagocytosis assay was used to assess the effect of H_2_ treatment on macrophage-mediated phagocytosis in A549 and H1975 cells (scale bar = 200 μm) (**P<0.01).

In addition, we investigated the role of H_2_ in lung cancer progression *in vivo* using the cancer tissues samples from our previous study [8]. As shown in the HE image, tumor cells were closely packed and cell atypia was obvious in the tumor tissues of the control group. Compared with the control group, tumor cell density and cell atypia was obviously decreased in both H_2_ and *cis*-platinum groups (Figure 5A). In addition, the expression levels of CD47 and CDC42 were decreased in both H_2_ and *cis*-platinum groups compared with the control group (Figure 5B). As CD47 is a regulator of macrophage-mediated phagocytosis [18], we conjectured that H_2_ might play a role in macrophage-mediated phagocytosis. Consistent to our assumption, we observed that the phagocytic index was significantly increased when A549 cells were administrated with 60% H_2_ in both A549 and H1975 cell lines (Figure 5C). These results further confirmed that H_2_ treatment repressed the progression of lung cancer via modulating CD47 expression.

## Discussion

Hydrogen presents mild reductive productivity with no toxicity in itself, which is beneficial to prevent and control serious toxicity side effects in medical procedures [19]. Since Dole et al. [7] put forward that H_2_ had the ability to treat cancers in 1975, Ohsawa et al. [6] found that Hydrogen had an anti-oxidant role which could selectively eliminate reactive oxygen species (ROS) in 2007. As to cancer, both increase and decrease in ROS levels all can break the redox homeostasis and bring redox stress, leading to cancer cell damage [20,21]. This discovery suggests that hydrogen exerts its anti-tumor role might via altering intratumoural ROS level. In addition, our research group found that H_2_ repressed the progression of lung cancer via regulating the expression of structural maintenance of chromosomes 3 (SMC3), which is an important regulator of chromosome condensation [8]. With the aim of further exploration of the mechanism of H_2_ in suppressing lung cancer progression, the present study illustrates that H_2_ represses the progression of lung cancer via down-regulating CD47.

In the current study, we observed that H_2_ treatment significantly decreased the expression levels of CD47 and CDC42. CD47, also termed as integrin-associated protein, is a 50-kDa cell surface glycoprotein and belongs to the immunoglobulin superfamily. CD47 is widely expressed on cell surface and serves as an antiphagocytic molecule via binding to signal regulatory protein α (SIRPα) [22,23]. CDC42 is a member of the Rho family of small GTPases and is activated by CD47 to serve as a crucial regulator of cancer metastasis [24]. Increasing evidences have shown that CD47 expression was elevated in tumor cancer tissues and cells, such as bladder cancer [25], leukemia [26], colon cancer [27] and NSCLC [28]. Its overexpression closely correlated with patients’ poor prognosis and significantly accelerated tumor cell transformation to malignant phenotypes. For example, Majeti et al. [26] reported that CD47 was highly expressed in acute myeloid leukemia (AML), and its increased expression level predicted shorter overall survival in adult AML patients. Moreover, blockage of CD47 with monoclonal antibodies enabled macrophage phagocytosis of AML leukemia stem cells and inhibited their tumorigenesis *in vivo*. Inhibition of CD47 with blocking antibodies or siRNA transfection obviously inhibited the migration of colon cancer SW480 cells in the presence of M2 macrophages [27]. Yoshida et al. [29] reported that CD47 was positively expressed in 49.6% (57/115) gastric cancer tissues, and its expression levels closely associated with the poor prognosis in gastric cancer. Taken together, CD47 is identified as a commonly expressed molecule on cancers, and blockade of its function leads to tumor cell phagocytosis and elimination. Therefore, CD47 is a proposed target for cancer therapies [30]. Here, we revealed that H_2_ treatment could negatively modulate CD47 expression, which might be the mechanism by which H_2_ inhibited lung cancer progression.

To further reveal CD47 roles in H_2_-mediated repression of lung cancer progression, we carried out the gain/loss-of-function assays. We observed that down-regulation of CD47 with si-CD47 transfection significantly enhanced H_2_ effects on the repressions of cell proliferation, invasion and migration, and the promotion of cell apoptosis. However, these above roles of H_2_ were apparently weakened when CD47 was overexpressed. These results indicate that H_2_ inhibits the progression of lung cancer via down-regulating CD47.

Zhang et al. [31] reported that blocking CD47 with a novel CD47-targeting fusion protein SIRPaD1-Fc led to a significant increase in macrophage-mediated phagocytosis in NSCLC cells, further confirming the antiphagocytic role of CD47 in lung cancer [28]. Based on this, we assume that H_2_ could also exert an antiphagocytic role in lung cancer cells. Consistently, we found that macrophage-mediated phagocytosis was significantly enhanced when cells were treated with H_2_ in both A549 and H1975 cells. Moreover, H_2_ administration could significantly alleviate the pathological change of lung cancer tissues from the *in vivo* mice, as well as cause obvious decreases in CD47 and CDC42 expressions.

In conclusion, the present study makes clear that H_2_ exerts an anti-tumor role in lung cancer via down-regulating CD47, an antiphagocytic molecule. As more and more attention has been attracted on the application of H_2_ therapy in clinical trials [32–34], H_2_ therapy should be proposed as a promising therapeutic strategy for cancer treatment.

## Abbreviations

AML: acute myeloid leukemia
CCK-8: cell counting kit-8
CDC42: cell division control protein 42
FBS: fetal bovine serum
HE: Hematoxylin and Eosin
H_2_: hydrogen gas
PARP: poly ADP-ribose polymerase
ROS: reactive oxygen species
RT-PCR: real-time PCR
siRNA: small interfering RNA
